# State-of-the-Art of Transcatheter Left Atrial Appendage Occlusion

**DOI:** 10.3390/jcm13040939

**Published:** 2024-02-06

**Authors:** Luigi Emilio Pastormerlo, Alberto Ranieri De Caterina, Augusto Esposito, Kasper Korsholm, Sergio Berti

**Affiliations:** 1 UOC Diagnostica Interventistica Fondazione Toscana Gabriele Monasterio Massa, 54100 Massa, Italy; pastormerlo@ftgm.it (L.E.P.); adecaterina@ftgm.it (A.R.D.C.); aesposito@monasterio.it (A.E.); 2 Department of Cardiology, Aarhus University Hospital, C319, 8200 Aarhus, Denmark; kasperkorsholm@clin.au.dk

**Keywords:** left atrial appendage, imaging, device, intracardiac echocardiography, transesophageal echocardiography, computed tomography

## Abstract

Left atrial appendage occlusion (LAAO) is an increasingly used alternative to oral anticoagulation in patients with atrial fibrillation, especially in patients with absolute/relative contraindications to these therapies. This review will cover three main aspects of the procedure. In the fist part of the manuscript, we focus on patient selection. We describe three main categories of patients with primary indication to LAAO, namely patients with previous or at a high risk of intracerebral bleeding, patients with a history of major gastrointestinal bleeding and patients with end-stage renal disease and absolute contraindication to novel oral anticoagulants. Some other potential indications are also described. In the second part of the manuscript, we review available devices, trying to highlight different aspects and potential specific advantages. The last section overviews different ways for pre-, intra- and postprocedural imaging, in order to improve procedural safety and efficacy and ameliorate patient outcome. The characteristics of available contemporary devices and the role of imaging in procedural planning, intraprocedural guidance and follow-up are described.

## 1. Patient Selection

### 1.1. Introduction

Atrial fibrillation (AF) is the most common cardiac arrhythmia with a prevalence exceeding 1800 per 100,000 in western Europe, with greater prevalence and incidence among the elderly and individuals with lifestyle-related comorbidities [1]. It is associated with up to one-fifth of all ischemic strokes, with annual stroke rates varying between 2% and more than 10% according to the baseline thromboembolic risk of the patient as estimated by their CHA_2_DS_2_VASc score [2]. Vitamin K antagonists (VKAs) reduce stroke by 60% and death by 25% compared with no antithrombotic treatment [3] but may be limited by contraindications in 10% according to historical series, while 2% may have an absolute contraindication [4,5]. Direct oral anticoagulants (DOACs) have consistently shown similar, or even improved, efficacy and a better safety profile compared to VKA [6,7,8,9,10]. Current guidelines favor DOAC over VKA as a first anticoagulation strategy in AF. However, barriers such as inadequate patient adherence, residual stroke or bleeding risks as well as economical limitations—in the US, the financial costs/insurance coverage limit the ability to take DOACs and a significant number of patients remain on VKA—limit optimal treatment. In phase 3 trials, discontinuation rates for DOAC ranged between 20 and 30% [6,7,8,9]. In clinical practice, nearly 20% of patients at risk of stroke do not receive any form of oral anticoagulation [11].

### 1.2. Left Atrial Appendage Occlusion for AF-Related Stroke Prevention

Left atrial appendage occlusion (LAAO) was developed as a direct alternative to anticoagulation, with the PROTECT-AF and PREVAIL trials showing that LAAO reduced the risk of all-cause mortality and major bleeding compared to warfarin [12,13]. Simultaneously with these results, the DOAC trials proved non-inferiority for stroke prevention with reduced bleeding risk compared to warfarin. This, in combination with a concern of procedural safety with LAAO, reported up to 10% in the PROTECT-AF cohort for any procedure-related event, led to an initial general concern for LAAO as a direct alternative to OAC. This skepticism remained despite the improved procedural success and safety over time.

Both European and American guidelines have remained cautious when considering LAAO as a primary choice of stroke prevention in patients with AF [14,15]. Presently, its indication is limited to patients who do not tolerate anticoagulation, with a IIb strength of recommendation and level of evidence B for ESC and, recently, IIa for AHA/ACC Guidelines [16]. However, the randomized PRAGUE-17 study in 2020 showed that LAAO was non-inferior to DOAC in preventing major AF-related cardiovascular, neurological and bleeding events in patients at a high risk of both stroke and bleeding [17]. Observational data support a low thromboembolic and hemorrhagic risk after LAAO, with a reduction in event rates compared to the predictions by CHA_2_DS_2_Vasc and HAS-BLED and comparable to DOAC [18,19,20,21,22,23]. In addition, in patients with anemia, while the risk-to-benefit ratio of assuming VKA or DOAC decreases, the stroke risk remains constant over the years irrespective of bleeding diathesis [24]. In this setting, LAAO represents a constantly underused strategy, also considering that the risk of experiencing a bleeding event is proportional to the duration of therapy. The longer you are treated with DOAC, the higher the risk of a serious bleeding event [25].

### 1.3. LAAO-Clinical Indications

A selection of patients for LAAO in clinical practice is difficult and more nuanced than currently provided by guidelines. How do clinical cardiologists best identify patients who will benefit from LAAO? Although the population with relative or absolute contraindication to OAC represents a minority of AF patients, this subgroup is numerically relevant worldwide. The term relative contraindication to OAC is vaguely defined in the literature, as is the definition of “high risk of bleeding” [26]. In clinical practice, it is not infrequent to discover patients managed with long-term low-molecular weight heparin or even antiplatelet therapy as a long-term thromboembolic strategy.

Patient evaluation should include a thorough investigation of bleeding history. Circumstances around the bleeding event may be inappropriate high INR levels, presence of transient conditions affecting the bleeding risk or reversible causes to the hemorrhagic event. Older patients are characterized by a higher incidence of OAC intolerance and, at the same time, may be at a higher risk of procedural complications [27]. In such cases, the continuation of oral anticoagulation or referral to LAAO should be carefully balanced. Available guidelines leave great uncertainty regarding the proper identification of patients with absolute or relative contraindication to oral anticoagulation. Below, we propose categories of patients who might benefit from a referral to LAAO (Table 1).

### 1.4. Patients with Previous Spontaneous Intracerebral and Intracranial Hemorrhage

For this category of patients, even if the first intracranial event happened on warfarin therapy, DOACs should be considered relatively contraindicated. Despite DOACs being associated with lower intracranial hemorrhage risk compared to warfarin [4,5,6,7], the residual risk of a second intracranial bleeding might be too high to consider the prescription of a permanent anticoagulant or antiplatelet therapy. In this context, LAAO was associated with a significant reduction in stroke/TIA and a remarkably low frequency of major bleeding during follow-up [17,28]. Among a small cohort of twenty-six patients—half of them with a history of symptomatic lobar hemorrhage—the LAA-CAA registry showed no major hemorrhagic events over a 25-month follow-up [29]. Two important randomized clinical trials, CLEARANCE (NCT04298723) and STROKECLOSE (NCT02830152), are currently enrolling patients with intracerebral or intracranial hemorrhage and AF, and are expected to provide more data on LAAO.

### 1.5. Patients at High-Risk of Intracranial Hemorrhage

Cerebral amyloid angiopathy (CAA) is characterized by the accumulation of amyloid beta-peptide within the leptomeninges and small/medium-sized cerebral blood vessels. The amyloid deposition results in fragile vessels that may manifest as lobar intracerebral hemorrhages (ICH). Approximately 12–15% of lobar ICH in the elderly is associated with CAA [30]. CAA is considered contraindicative to long-term DOAC by most neurologists, and referral for LAAO appears suitable as the equipoise of treatment strategies has been lost by physicians.

### 1.6. Patients with Previous Major Gastrointestinal Bleeding

Approximately 10 to 20% of GI bleedings cannot be definitively corrected, thus potentially representing a continuous risk of harm over time [31]. Patients presenting with GI bleeding without a reversible cause (e.g., diffuse angiodysplasia, diverticular disease) may benefit from LAAO. The recent AHA/ACC specifically includes bleeding due to a non-reversible gastrointestinal focus as a contraindication towards long-term OAC [16]. The clinical applicability and positive effect of LAAO on stroke and bleeding rates were demonstrated by Lempereur et al., demonstrating a relative risk reduction by 20% compared to the HAS-BLED score [32]. However, most evidence is based on non-randomized data. The randomized ASAP-TOO trial (NCT02928497) included such patients, among other indications, but was terminated prematurely due to slow enrolment and a concern among investigators of lacking equipoise. Conversely, LAAO may imply antithrombotic treatment in the postprocedural period. Major bleeding (most often GI) is a common complication after LAAO. Hence, the composition and duration of post-LAAO antithrombotic treatment needs consideration, and the option of leaving the patient without antithrombotic therapy in the first 3 to 6 months after LAAO has been described as a potential viable solution, but warrants confirmation in larger, prospective studies.

### 1.7. Patients with End-Stage Renal Disease or on Haemodialysis

In patients with AF and end-stage renal disease or on hemodialysis—a population at a particularly high risk of embolic stroke—warfarin control is poor and may even be associated with rapid worsening of renal function secondary to warfarin-related nephrocalcinosis. Additionally, most DOACs are contraindicated, although observational data may show promise. In a cohort of 132 patients, the replacement of VKAs by rivaroxaban—at a dose of 10 mg daily—was safe and potentially associated with less life-threatening and major bleeding [33]. Conversely, using apixaban, while the RENAL-AF was prematurely stopped due to inadequate power to draw any conclusion, showing clinically relevant bleeding events were ≈10-fold more frequent than stroke or systemic embolism [34]; the AXADIA-AFNET 8 revealed no differences in safety or efficacy outcomes between apixaban and warfarin [35]. In any case, current guidelines contraindicate the use of DOACs in patients with end-stage renal disease or on hemodialysis [36]. In the setting of LAAO, it should be acknowledged that renal failure is associated with a higher risk of DRT after LAAO [37]. The ongoing LAA-KIDNEY trial (NCT05204212) will provide specific periprocedural and long-term outcomes in this category of patients.

Beside these major categories of patients, LAAO might be considerable in patients with

- ***Hematologic disorders*** with a relative contraindication to antithrombotic therapy, such as myelodysplastic disorders, thrombocytopenia or hemophilia [38].
- Those patients for whom prolonged (and even double) antiplatelet therapy would be advisable, such as those with multiple coronary stents, multiple previous recurrent acute coronary syndromes or cerebral events secondary to carotid disease. Here, the combination of oral anticoagulation and antiplatelet therapy might result in a too-high long-term bleeding risk. In this specific category of patients, careful balancing risk factors and a multidisciplinary discussion including patient’s preference should all be taken into account when considering stroke prevention strategies.
- ***Recurrent stroke despite optimal anticoagulation.*** The potential indication for LAAO is represented by patients who experienced an ischemic stroke despite optimal anticoagulant therapy, then other plausible causes (e.g., carotid disease, severe mobile aortic arch atheromata) are excluded. ESC guidelines in this context recommend optimization of anticoagulant therapy [39] while adding an antiplatelet agent to OAC is another practice that may be encountered in the clinical arena, even if there are no available data supporting this approach. Seiffge et al. showed that the risk of stroke recurrence was very high (8–9%/yr) and a change of OAC strategy did not change the risk of stroke recurrence [38]. LAAO may be considered in this population as an alternative stroke preventive measure. Observational propensity-matched studies suggest a significantly lower risk of the composite outcome of stroke, major bleeding and all-cause mortality with LAAO therapy compared to DOAC but warrants confirmation in randomized trials [20,40]. Currently, two randomized clinical trials enroll patients within this category and compare outcomes to DOAC therapy; the OCCLUSION-AF trial (NCT03642509) randomizing between DOAC and LAAO combined with long-term SAPT, and the ELAN trial (NCT05976685) randomizing between DOAC and LAAO combined with continued DOAC-therapy.
- ***Non-compliant patients****,* including those not willing to take medications at all, those subjects with a specific lifestyle or profession leading to no or irregular drug intake or those presenting with non-compliance despite multiple measures to improve this. In any case, according to ESC guidelines, these patients should be strongly encouraged to take DOACs, which still represent the first option.

Given the continuously improving safety of the procedure and cumulating evidence of its long-term efficacy, LAAO deserves consideration in the presence of intolerance to OAC or high-hemorrhagic risk. Ongoing trials will provide high-level evidence within the coming years, including trials comparing LAAO against DOAC in a broad AF population with CHA2DS2-VASc > 2. The above-mentioned patient categories for whom LAAO should be considered should be considered as a tool to help clinical cardiologists manage AF-patients in daily practice. The decision between treatment strategies should be based on a multidisciplinary approach, considering the patient perspective as reflected in the most recent EAPCI/EHRA consensus statement [41].

## 2. Contemporary Devices

LAAO may be achieved by various devices with the CE Mark, while the Watchman FLX and Amplatzer Amulet are currently the only approved devices with both CE Mark and Food and Drug Administration (FDA) approval. LAAO devices are categorized as epicardial or endocardial, with the latter consisting of devices categorized as either plug-based or disc-lobe platforms [42]. Here, endocardial devices will be discussed (Table 2).

### 2.1. The Watchman Device

**Figure 1:**
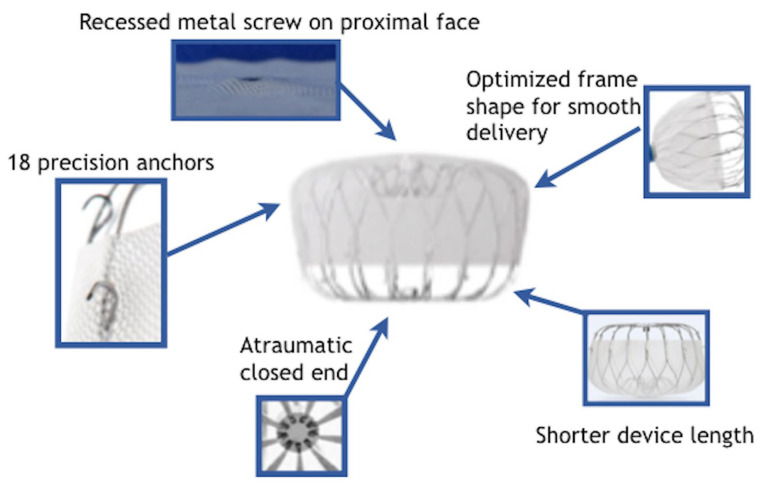
Novel characteristics of the Watchman Flex Device in comparison to previous versions.

The Watchman 2.5 device (Boston Scientific Corporation, Boston, MA, USA) was evolved based on the first PLAATO device dedicated to LAAO. It consisted of a self-expandable, parachute-shaped nitinol device. The first device in the Watchman family was the Watchman 2.5., which received CE Mark approval in 2005 and US Food and Drug Administration (FDA) approval in 2015. The Watchman 2.5 successor is the Watchman Flx (Boston Scientific Corporation). It features a closed-end self-expanding nitinol material with a parachute shape, 18 strut structures and 24 J-shaped anchors. The closed end renders it atraumatic while a more distal extension of the polyethylene terephthalate membrane fabric is designed to achieve a better seal and reduce peri-device leakage (PDL). It is available in five different sizes (20, 24, 27, 31 and 35 mm) for LAA ostia ranging between 14 and 32 mm. It can be completely recaptured, repositioned and reinserted for optimal positioning. Watchman Flx novel characteristics in comparison to previous Watchman devices are summarized in Figure 1. The device is implanted with a 14 Fr Watchman FXD Curve sheath delivery system. This delivery system is available in three curvatures in order to accommodate various access angulation between transseptal puncture, left atrium and LAA orientation. The Watchman device is the most studied of all the LAA closure devices both for safety and for clinical efficacy. The most robust data for the safety and clinical effectiveness of the Watchman device in comparison with oral anticoagulation are available from the two FDA registration trials—PROTECT-AF and PREVAIL. Refs. [12,13] explored the direct comparison between LAAO and oral anticoagulation with warfarin while the EWOLUTION is one of the widest registries of LAAO procedures for patients with absolute/relative contraindication to OAC [43]. Compared to the Watchman 2.5 device, the Watchman FLX was associated with lower rates of in-hospital MAE including mortality, pericardial effusion, major hemorrhage, cardiac arrest, and device embolization [44,45,46]. The latest generation of Watchman devices features a fluoropolymer-coated membrane (polyvinylidenefluoride-co-hexafluoropropylene), which should increase thromboresistance, facilitate tissue ingrowth and hence reduce the risk of device-related thrombosis [47].

### 2.2. Amplatzer Devices

The Amplatzer Amulet occluder device (Abbott, Abbott Park, IL, USA) is the uploaded version of the earlier Amplatzer Cardiac Plug device. It is a self-expanding nitinol device that consists of a lobe and a disc connected by a central waist. Polyester patches are sewn into both the lobe and disc to facilitate occlusion. The lobe-and-disk platform appears to be more functional in cases of left atrial appendages with little implantation depth and in cases of anatomies in which the axis of the ostium and body of the appendage are arranged and oriented in different angles. In such cases, the Amulet appears to be a very effective device with good sealing and low incidence of periprocedural complications [48,49]. Device sizing is evaluated according to the so-called landing zone of the device lobe that is considered as a 10 to 12 mm line perpendicular to the ostium plane inside the LAA. Eight different sizes ranging from 16 to 34 mm are available to cover LAA landing zone width from 11 to 31 mm. The Amulet device is implanted through a 12 (16 to 25 mm devices) or 14 Fr (28–34 mm devices) double-curved TorqVue sheath [50].

In the Amulet IDE randomized trial (*n* = 1878), the Amulet occluder was non-inferior for the safety and effectiveness of stroke prevention compared with the Watchman device [50]. Occlusion rates of LAA were slightly higher with Amulet (98.9% versus 96.8%) even if the procedure-related complications rate was higher compared with the Watchman device (4.5% versus 2.5%). The SWISS APERO trial randomized 221 patients to either Amulet or Watchman (77.3% patients received Watchman Flx). The rate of residual LAA patency was similar between Amulet and Watchman at a 45-day cardiac CT. Still, Amulet was associated with lower PDL rates on a transesophageal echocardiography (TEE) and had similar clinical outcomes at 45 days compared with Watchman. On the other side, a slightly higher rate of pericardial effusion/pericardial tamponade was reported with the Amulet device in the SWISSS APERO trial [51]. Further studies comparing the Amulet device with Watchman Flx are awaited.

### 2.3. Ultraseal Device

The Ultraseal LAA occluder represents the second generation of Cardia. Similar to other devices, it is a self-expandable distal nitinol lobe, with a proximal disc and an articulating communicating waist. It is available in 10 sizes, ranging from 16 to 34 mm, with lengths between 10 and 18 mm. The device features a reduced radial force and more flexible central waist between the disc and lobe. This results in a more compact device capable of adapting to more complex anatomies. An oversizing of 10–20% between the lobe and the landing zone is recommended [51].

At present, limited data are available, but in-hospital complication rates have been reported at around 5.8% in a multi-center experience across seven European centers and 52 patients [52]. Two-thirds of the patients underwent a follow-up TEE after 61 days with PDL > 5 mm in 2.9% of patients, while no patients had a device-related thrombus (DRT) [52].

### 2.4. OMEGA Device

The OMEGA© LAAO device is an extruded nitinol (288-thread) double-layer metal mesh extrusion coated with inert platinum, and consists of the following main parts: distal cup, proximal disc with polypropylene filling and polyester thread stitching, connecting strap, anchoring hooks in pairs of staggered geometry in increasing numbers according to the size of the device and proximal female connecting joints and thread embedded in the disc [53].

Furthermore, the Omega LAA Occluder device has a size range from 14 mm to 30 mm, making its smallest size (14 mm) unique on the market, which enables treatment from landing zones as small as 10 mm and thus making it the LAA device capable of treating very small LAA. There are currently 500 cases in Europe, but without a dedicated registry [54,55].

### 2.5. Lambre Device

The Lambre device (Lifetech Scientific, Shenzhen, China) is a self-expanding, nitinol-based device with a hook-embedded umbrella and a cover connected with a short central waist. The proximal cover is larger in diameter than the umbrella, sewn with polyethylene terephthalate fabric and intends to cover the LAA orifice on deployment. The distal umbrella comprises eight claws with individual stabilizing hooks, as well as a polyethylene terephthalate membrane. The device comes in several sizes ranging from an umbrella diameter of 16–36 mm [56]. The most important distinguishing feature for Lambre is the availability of “special” devices with large covers for small umbrellas (e.g., umbrella/cover of 22/34 or 26/38 mm). This could facilitate implantation in “chicken wing” anatomies with a very short implantation zone or conical LAAs with a large size difference between the ostium and the medial part of the LAA. Still, the double stabilization system may potentially reduce embolization and facilitate implantation by reducing the number of recaptures.

### 2.6. CLAAS Device

The CLAAS device (Conformal Left Atrial Appendage Seal; Conformal Medical, Nashua, NH, USA) is a foam-based, self-expanding occluder consisting of a cylindrical nitinol endoskeleton covered with a conformable, porous, polyurethane-carbonate matrix foam. The endoskeleton is stabilized by two anchoring wires arranged in two parallel rows. The device is available in only two sizes: 27 mm for an average LAA diameter between 13 and 25 mm and 35 mm for an average LAA diameter between 20 and 32 mm [57]. In a preclinical study of device implantation in seven healthy male canines in sinus rhythm, histological examination at 60 days showed complete neointima coverage with minimal inflammation. This first-in-human study demonstrates the clinical feasibility of the CLAAS device for LAAC [58].

### 2.7. New Devices

Several other devices are under development. The WAVECREST 2 trial is ongoing in the USA. The randomized controlled trial was designed to ensure the safety and effectiveness of the WaveCrest device (Biosense Webster, Irvine, CA, USA) in comparison to the Watchman device. The WaveCrest device consists of a self-expanding nitinol frame with 20 anchoring points, covered by an expanded polytetrafluoroethylene. Some potential advantages are related to safer repositioning, occlusive and non-thrombogenic device material, no metal exposed on the left-atrium-facing surface, potential for distal contrast injection, design for a short landing zone and device seal on the distal margin of the device.

Many other devices are under pre-/clinical evaluation such as SeaLA LAA occluder (Hangzhou Valued Medtech, Hangzhou, China), LACbes (PushMed), Occlutech LAA Occluder (Occlutech, Helsingborg, Sweden) and Laminar device (Laminar, Inc., Santa Rosa, CA, USA) [59,60].

## 3. Procedural Planning and Execution

**Figure 2:**
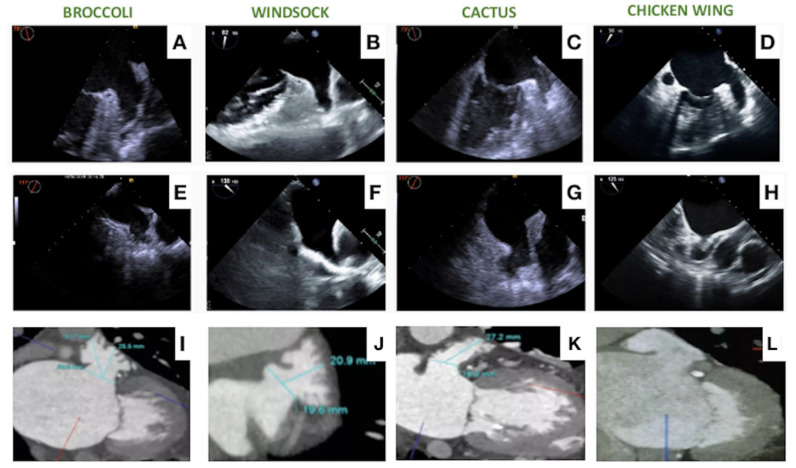
Most common LAA anatomies as visualized by TEE and CT, i.e., Broccoli (**A**,**E**,**I**), Windsock (**B**,**F**,**J**), Cactus (**C**,**G**,**K**), Chicken Wing (**D**,**H**,**L**).

**Figure 3:**
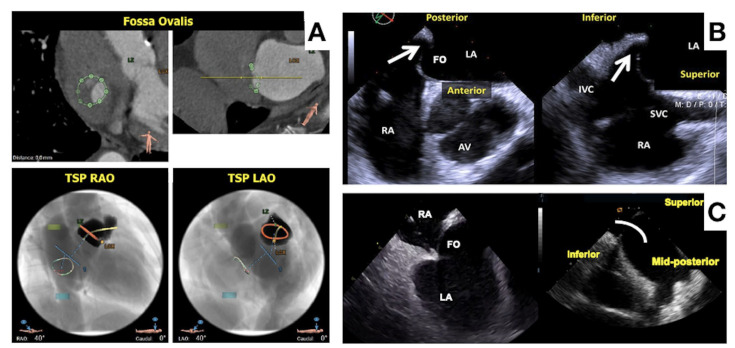
(**A**): CT planning for optimized transeptal puncture in superoinferior and antero-posterior planes; (**B**): transeptal puncture with TEE imaging; (**C**): transeptal puncture with ICE imaging.

LAAO represents a complex intervention, mainly due to the anatomical complexity of the LAA with a high degree of heterogeneity among patients. Several classification systems exist to help categorize LAA anatomies, with the most commonly used classifying the LAA into chicken wing, windsock, cactus and broccoli morphologies (Figure 2). In real life, classification often simplifies the anatomical complexity, and a significant overlap between morphologies exist. Other variables that may significantly affect the LAAO procedure are the presence of extensive trabeculation, multiple lobes that may be located proximally and the thin-wall LAA structure that may predispose to pericardial effusion. The access to the LAA may influence the ability to achieve a coaxial alignment of the LAAO device with the LAA wall. Here, the anatomy of the interatrial septum and fossa ovalis may vary in terms of thickness and width, creating a challenge for transeptal puncture (Figure 3). An inferior and posterior puncture is considered most optimal to engage the LAA, but in select cases such as retroverted chicken wing morphologies, a more inferior mid or anterior puncture may be advisable [61]. Finally, the left atrial volume and spatial relationship of the LAA to surrounding structures may affect the possibility to access the LAA and position a device.

Imaging is paramount to achieve an optimal result. Both transesophageal echocardiography (TEE) and cardiac computed tomography (CCT) may be used in the preprocedural planning and postprocedural follow-up. Intraprocedural guidance has relied on TEE, but intracardiac echocardiography (ICE) is a valid alternative to TEE. LAAO operators should be familiar with the interpretation and incorporation of imaging results, which are crucial to gain proficiency of this intervention. In complex cases, different imaging modalities may be complementary.

Preprocedural has a crucial role to ensure procedural safety and efficacy. Excluding absolute/relative contraindications such as LAA thrombosis is mandatory in the preprocedural phase. Preprocedural imaging may affect the choice of device and its size for that specific anatomy and provide important procedural details as the best position for transeptal puncture and implant view. Three-dimensional TEE is widely used and considered the gold-standard modality for preprocedural evaluation. CCT is a valid alternative and may provide a more accurate assessment of the LAA geometry and dimensions due to higher spatial resolution. Moreover, CCT is less invasive than TEE, highly efficient with a more reproducible evaluation and off-line interpretation. 3D volume rendering provides a roadmap for transseptal puncture (Figure 3) and optimal C-arm angulations for device implantation, which may reduce the amount of contrast used during the procedure [62]. Additionally, CCT imaging may be used for 3D printing, simulation and computational modeling, as well as for fusion/overlap imaging.

Both TEE and CCT have advantages and limitations, as summarized in Table 3. CCT is limited by contrast medium use, radiation exposure, availability and costs, while TEE is limited by the need for fasting and sedation with associated patient discomfort and is more operator dependent.

Intraprocedural echocardiography has documented benefits compared to fluoroscopic guidance alone [63]. It serves as guidance for transseptal puncture, navigation of sheaths inside the left atrium and LAA, positioning of the device at the intended landing zone and securing adequate device placement and sealing. Intraprocedural imaging is crucial for the avoidance/early detection of procedural complications such as pericardial effusion, procedure-related stroke device embolization or impingement of surrounding structures. TEE is still most widely used. The advantages include high-quality 2D and 3D images with multiplane capabilities. Limitations include requirements for sedation, an additional TEE-operator and dedicated anesthesia staff present. Furthermore, the risk of esophageal injury in TEE-guided cardiac interventions is more significant than previously anticipated [64]. ICE represents an increasingly used alternative. The main conceptual difference between TEE and ICE is the probe position. The TEE probe is placed in the esophagus for viewing the anatomical structures of the heart from a position behind the LA, while the ICE probe is advanced, usually via a femoral vein approach directly into the heart for viewing the target anatomical structures directly from a heart chamber or a large vessel (right atrium, left atrium, pulmonary artery, etc.). (Table 4) ICE appears non-inferior to TEE for guiding LAAO in terms of procedural success, peri-procedural complications and long-term patients’ outcome [65,66,67,68,69]. ICE may even be superior to TEE in the imaging of the fossa ovalis (Figure 3) and in the imaging of clots/vegetations on pacemaker leads, as well as for some specific LAA anatomies. Most currently available ICE probes are limited by 2D imaging, but 3D ICE probes with digital multiplane capabilities are now becoming available (FDA approved) which may provide further support to the ICE approach [70]. At present, 3D preprocedural planning (by CCT or TEE) is considered a prerequisite for successful ICE-guided LAAO.

The ICE position in the left atrium provides the most optimal intraprocedural imaging. Three main projections have been described for LAA evaluation and procedural guidance: the left upper pulmonary vein view, the mid-atrial view and the supramitral view. These three views are equivalent to the most common TEE views, respectively, 45°, 90° and 135° projections.

**Figure 4:**
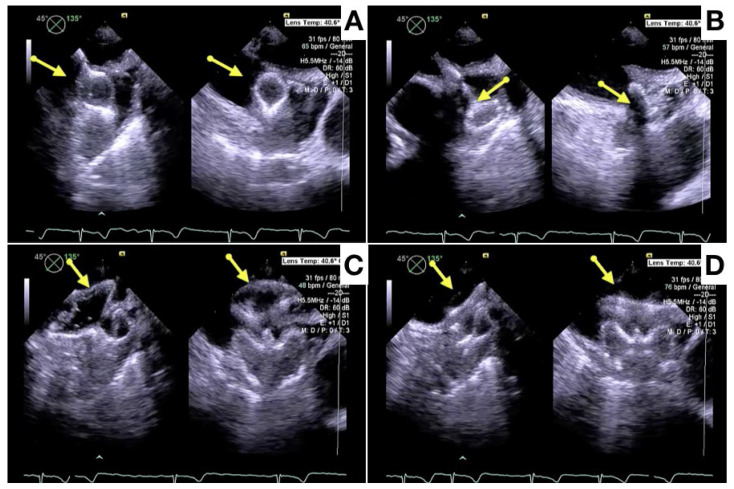
Different phases of Amulet device release with x-plane TEE imaging: lobe release (**A,B**), traction test after disc release (**C**), final position of the device (**D**).

**Figure 5:**
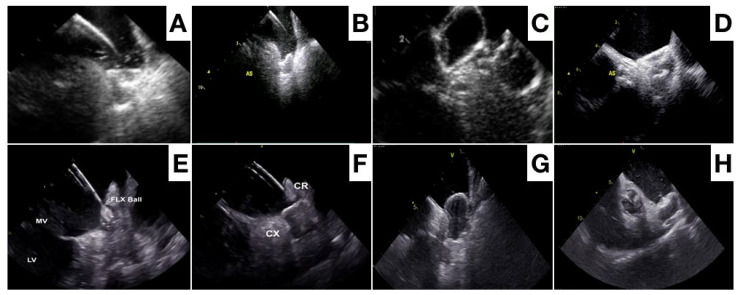
Different phases of Amulet device (**A**–**D**) and of Watchman FLX device (**E**–**H**) with ICE imaging.

**Figure 6:**
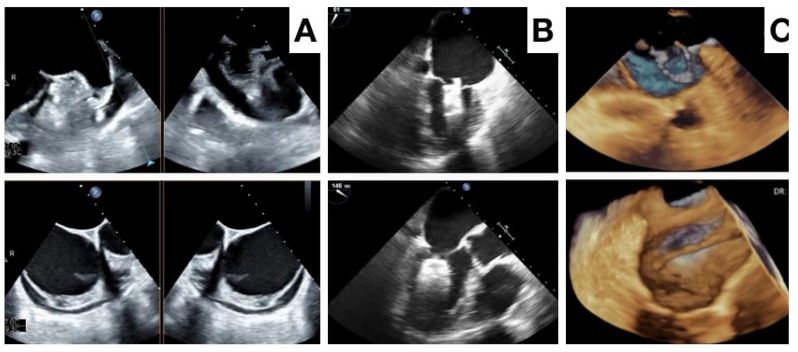
Potential intraprocedural complications as visualized by TEE, pericardial effusion in (**A**), device embolization in (**B**), device acute thrombosis in (**C**).

After device deployment, the device position relative to the specific anatomical landmarks of the circumflex artery and left upper pulmonary vein is evaluated. The device morphology and compression, along with absence of a peridevice leak needs confirmation before device release (Figure 4 and Figure 5). Although both ICE and TEE provide sufficient imaging information to evaluate this [64,65,66,67,68], a 3D imaging modality may be superior for a position assessment and (small) peridevice leak exclusion. After device release, the device position and peridevice leak are confirmed, and complications like pericardial effusion excluded (Figure 6).

## 4. Follow-Up

Follow-up device surveillance remains crucial in the recognition of complications like device-related thrombosis (DRT) and peridevice leak (PDL). Significant heterogeneity exists in the timing of imaging follow-up across centers and studies. Early imaging follow-up is recommended between 45 and 90 days after LAAO, while the evidence supporting repeated, later imaging is weak and mainly performed in cases of DRT or significant PDL at early imaging.

**Figure 7:**
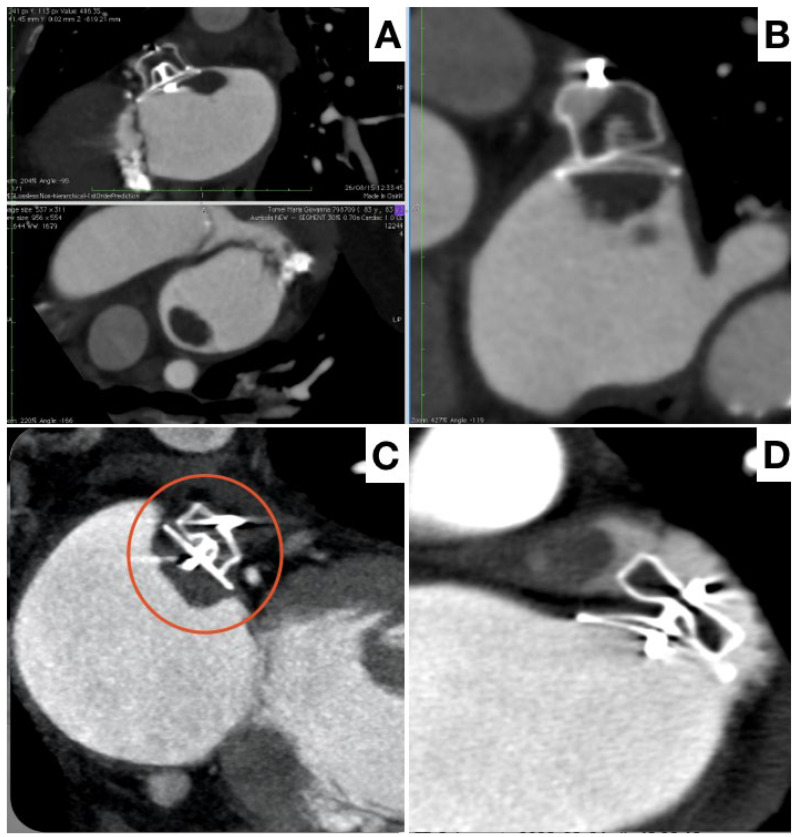
Different potential aspects of thrombosis at follow-up by CT. Pedunculated, hypermobile thrombus attached to the device (**A**), flat and stratified thrombus on the device (**B**,**C** red circle), presence of thrombus within unexcluded LAA (**D**).

The incidence of DRT is reported around 3% to 4%, but with significant variation associated with the imaging modality used, timing of follow-up and number of scans performed. Multiple studies have documented an association between DRT and ischemic events [71,72,73]. Nevertheless, the management of DRT is challenged by a lack of reproducible diagnostic criteria and optimal detection protocols. Despite TEE offering higher temporal resolution, cardiac CT has superior spatial resolution, is isotropic by nature with three-dimensional (3D) and multiplanar capabilities and is less operator-dependent. CCT seems more sensitive for DRT detection and may give information about its exact position and size, as well as on its base of implant and stability (Figure 7).

Standardized CCT classifications of DRT have been developed which rely on the documentation of hypoattenuated thickening (HAT) on the device surface cap, with HAT < 3 mm defined as low grade (the clinical outcome of which is uncertain), and HAT > 3 mm defined as high grade and diagnostic of DRT [73].

Various predictors associated with DRT have been identified. Many of them are non-modifiable factors such as such as hypercoagulability disorder, non-paroxysmal AF, renal insufficiency, previous stroke and a generally elevated CHA2DS2-VASC score. From a technical perspective, an implantation depth > 10 mm from the pulmonary vein limbus has been associated with higher DRT risk, and may be modifiable during the intervention. This result underscores the key role of intraprocedural imaging [74].

DRT diagnosis is inherently linked to the imaging regimen pursued, with the empirical concept of early and late imaging appearing justified, while clinically driven imaging should be performed (i.e., following an embolic event). Implementing unbiased standardized surveillance protocols and core laboratory evaluation of cases based upon specific modalities and definitions would be beneficial to obtain insights into DRT timing, device-specific considerations, optimal follow-up regimens and into DRT persistence or recurrence. The choice between CCT and TEE in the need for multiple re-evaluations is affected by intrinsic limitations such as contrast medium use and radiation exposure for CCT, and invasiveness and discomfort associated with a low patient tolerance for TEE.

**Figure 8:**
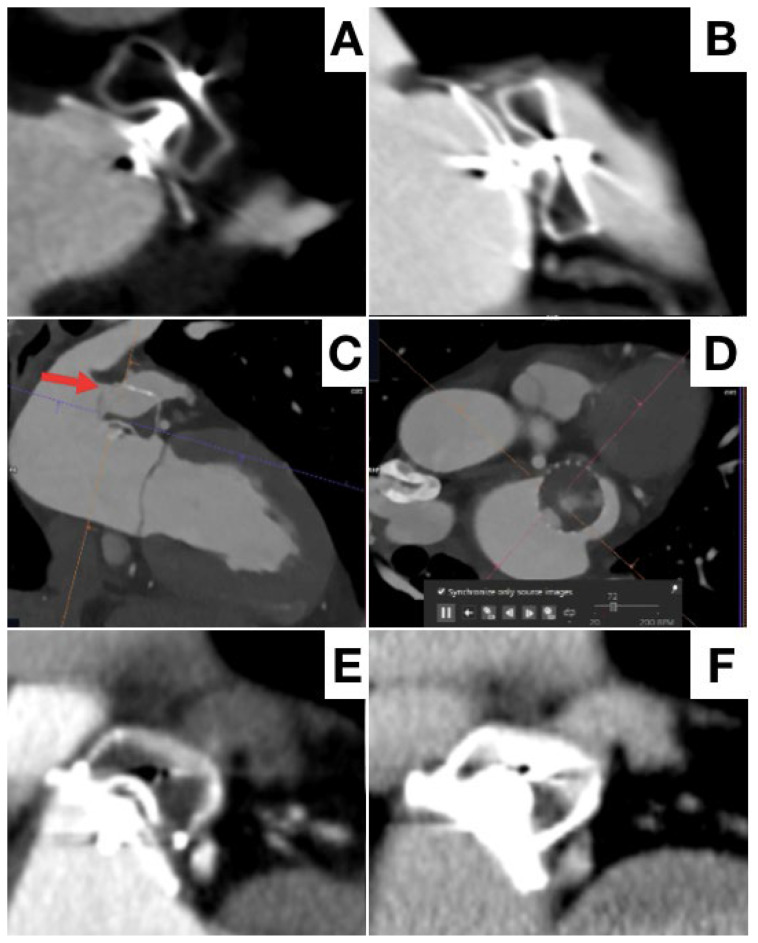
Different potential aspects of peridevice leak at follow-up by CT. No leak (**A**), high flow peridevice leak due to incomplete LAA occlusion (**B**), high flow-leak due to device malposition and rotation (**C**,**D**), low-flow leak only in venous phase (**E**,**F**).

The second Achilles’ heel of LAAO during follow-up is residual PDL, indicating that the LAA is only partially excluded from general circulation. PDL after LAAO are common, but the incidence varies considerably depending on applied definitions and imaging modality (higher with CCT) [75]. An arbitrary cut-off of 3 or 5 mm PDL diameter to define clinically significant leaks has been used for TEE. Various CCT classifications have been applied across studies, but is based on the principles of contrast opacification in the distal LAA (contrast patency), sealing of the device (PDL) and definition of the potential mechanism of residual leak [76,77] (Figure 8).

Despite conflicting results, the large NCDR-LAAO Registry recently reported an association between PDL (<5 mm) and a modest but statistically significant increased risk of stroke/transient ischemic attack/systemic embolization at 1 year [78].

## 5. Final Considerations

LAAO for stroke prevention in AF is growing worldwide. Patient selection is crucial, with guidelines currently recommending consideration of LAAO in OAC contraindicated patients. Ongoing clinical trials will provide evidence on its use in specific patient categories but also will evaluate LAAO as a direct alternative to OACs. For this purpose, procedural safety and efficacy must be maximized by device technology improvements and peri-procedural imaging.

The Watchman and Amulet devices are the two most widely used for LAAO, with best supporting evidence. Several new devices are on the runway. Preprocedural, intraprocedural and follow-up imaging are increasing safety and maintain immediate and long-term efficacy of the LAAO intervention.

## Figures and Tables

**Table 1:** Clinical indications for LAAO procedure.

**Primary Indications**	
	Previous intracranial hemorrhage
	High risk of intracranial hemorrhage
	Previous major GI bleeding
	End-stage renal disease
**Potential Indications**	
	Hematologic disorders
	Advisable prolonged anti-platelet therapy
	Recurrent events despite optimal anticoagulation
	Non-compliant patients
	Young patients (<55 years old, CHA_2_DS_2_-VASC > 1)

**Table 2:** Contemporary devices for LAAO procedure.

Device	Imagine	Design	Sizes (MM)	Sheat (F)	Company	Status CE
**Watchman 2.5**	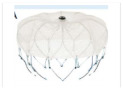	Single (lobe)	21, 24, 27, 30, 33	14	Boston Scientific Corporation	CE Mark (2005)
**Watchman FLX**	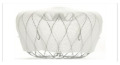	Single (lobe)	20, 24, 27, 31, 35	14	Boston Scientific Corporation	CE Mark (2015)
**Amplatzer cardiac Plug**	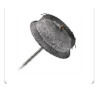	Double (lobe and disc)	16, 18, 20, 22, 24, 26, 28, 30	9, 13	Abbott Vascular	CE Mark (2008)
**Amplatzer Amulet**	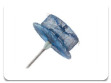	Double (lobe and disc)	16, 18, 20, 22, 25, 28, 31, 34	12, 14	Abbott Vascular	CE Mark (2013)
**Ultraseal**	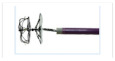	Double (bull and sall)	16, 18, 20, 22, 24, 26, 28, 30, 32	10, 12	Cardia, Inc.	CE Mark (2016)
**Omega**	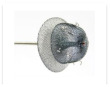	Double (lobe/cup and disc)	14, 16, 18, 20, 22, 24, 26, 28, 30	14	Eclipse Medical	CE Mark (2021)
**LAmbre**	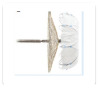	Double (umbrella and cover)	16, 18, 20, 22, 24, 26, 28, 30, 32, 34, 36	8, 10	Lifetech Scientific, Co., Ltd.	CE Mark (2016)
**CLAAS**	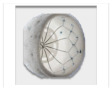	Adaptable form	27, 35	17	Conformal Medical	Non approved

**Table 3:** Comparison of limits and advantages of CCT vs. TEE for preprocedural planning of LAAO.

	TEE	CCT
**ADVANTAGES**		
	Standard of care in many centers	Higher spatial resolution
	No contrast medium	Low inter-observer variation
	No radiations	Optimal transeptal puncture planning
	Lower cost	Implant view planning
	Easily available	Potential for simulation/modeling
**LIMITS**		
	Invasive	Contrast medium use
	Gastro-esophageal contraindications	Radiations
	Not optimal imaging in some cases	Higher cost

CCT: cardiac computed tomography; LAAO: left atrial appendage occlusion; TEE: transesophageal echocardiography.

**Table 4:** Comparison of limits and advantages of TEE vs. ICE for intra-procedural imaging of LAAO.

	TEE	ICE
**ADVANTAGES**		
	Standard of care in many centers	No need for orotracheal intubation
	Many cardiologists are familiar with TEE	Local anesthesia
	3D evaluation	Better visualization of LAA from LA
	Higher quality	No need for dedicated operator
	Easily available	
	Low cost	
	Possible re-evaluation in the cathlab before vascular access	
**LIMITS**		
	Invasive	2D only (need for a preprocedural 3D evaluation)
	Gastro-esophageal contraindications	Higher use of contrast medium
	Not optimal imaging in some cases	Higher cost
	Interference with fluoroscopy	Limited operator experience
		Positioning in LA mandatory for good quality

ICE: intracardiac echocardiography; LAAO: left atrial appendage occlusion; TEE: transesophageal echocardiography.

## References

[1] Go A.S., Hylek E.M., Phillips K.A., Chang Y., Henault L.E., Selby J.V., Singer D.E. (2001). Prevalence of diagnosed atrial fibrillation in adults: National implications for rhythm management and stroke prevention: The anticoagulation and risk factors in atrial fibrillation (ATRIA) Study. JAMA.

[2] Flegel K.M., Shipley M.J., Rose G. (1987). Risk of stroke in non-rheumatic atrial fibrillation. Lancet.

[3] Hart R.G., Pearce L.A., Aguilar M.I. (2007). Meta-analysis: Antithrombotic therapy to prevent stroke in patients who have nonvalvular atrial fibrillation. Ann. Intern. Med..

[4] O’Brien E.C., Holmes D.N., Ansell J.E., Allen L.A., Hylek E., Kowey P.R., Gersh B.J., Fonarow G.C., Koller C.R., Ezekowitz M.D. (2014). Physician practices regarding contraindications to oral anticoagulation in atrial fibrillation: Findings from the Outcomes Registry for Better Informed Treatment of Atrial Fibrillation (ORBIT-AF) registry. Am. Heart J..

[5] Steinberg B.A., Greiner M.A., Hammill B.G., Curtis L.H., Benjamin E.J., Heckbert S.R., Piccini J.P. (2015). Contraindications to anticoagulation therapy and eligibility for novel anticoagulants in older patients with atrial fibrillation. Cardiovasc. Ther..

[6] Connolly S.J., Ezekowitz M.D., Yusuf S., Eikelboom J., Oldgren J., Parekh A., Pogue J., Reilly P.A., Themeles E., Varrone J. (2009). Dabigatran versus warfarin in patients with atrial fibrillation. NEJM.

[7] Patel M.R., Mahaffey K.W., Garg J., Pan G., Singer D.E., Hacke W., Breithardt G., Halperin J.L., Hankey G.J., Piccini J.P. (2011). Rivaroxaban versus warfarin in nonvalvular atrial fibrillation. NEJM.

[8] Granger C.B., Alexander J.H., McMurray J.J., Lopes R.D., Hylek E.M., Hanna M., Al-Khalidi H.R., Ansell J., Atar D., Avezum A. (2011). Apixaban versus warfarin in patients with atrial fibrillation. NEJM.

[9] Giugliano R.P., Ruff C.T., Braunwald E., Murphy S.A., Wiviott S.D., Halperin J.L., Waldo A.L., Ezekowitz M.D., Weitz J.I., Špinar J. (2013). Edoxaban versus warfarin in patients with atrial fibrillation. NEJM.

[10] Ruff C.T., Giugliano R.P., Braunwald E., Hoffman E.B., Deenadayalu N., Ezekowitz M.D., Camm A.J., Weitz J.I., Lewis B.S., Parkhomenko A. (2014). Comparison of the efficacy and safety of new oral anticoagulants with warfarin in patients with atrial fibrillation: A meta-analysis of randomised trials. Lancet.

[11] Kakkar A.K., Mueller I., Bassand J.P., Fitzmaurice D.A., Goldhaber S.Z., Goto S., Haas S., Hacke W., Lip G.Y., Mantovani L.G. (2013). Risk profiles and antithrombotic treatment of patients newly diagnosed with atrial fibrillation at risk of stroke: Perspectives from the international, observational, prospective GARFIELD registry. PLoS ONE.

[12] Holmes D.R., Reddy V.Y., Turi Z.G., Doshi S.K., Sievert H., Buchbinder M., Mullin C.M., Sick P. (2009). PROTECT AF Investigators. Percutaneous closure of the left atrial appendage versus warfarin therapy for prevention of stroke in patients with atrial fibrillation: A randomised non-inferiority trial. Lancet.

[13] Holmes D.R., Kar S., Price M.J., Whisenant B., Sievert H., Doshi S.K., Huber K., Reddy V.Y. (2014). Prospective randomized evaluation of the Watchman Left Atrial Appendage Closure device in patients with atrial fibrillation versus long-term warfarin therapy: The PREVAIL trial. J. Am. Coll. Cardiol..

[14] January C.T., Wann L.S., Alpert J.S., Calkins H., Cigarroa J.E., Cleveland J.C., Conti J.B., Ellinor P.T., Ezekowitz M.D., Field M.E. (2014). 2014 AHA/ACC/HRS guideline for the management of patients with atrial fibrillation: A report of the American College of Cardiology/American Heart Association Task Force on practice guidelines and the Heart Rhythm Society. Circulation.

[15] Kirchhof P., Benussi S., Kotecha D., Ahlsson A., Atar D., Casadei B., Castella M., Diener H.C., Heidbuchel H., Hendriks J. (2016). 2016 ESC Guidelines for the management of atrial fibrillation developed in collaboration with EACTS. Eur. Heart J..

[16] Joglar J.A., Chung M.K., Armbruster A.L., Benjamin E.J., Chyou J.Y., Cronin E.M., Deswal A., Eckhardt L.L., Goldberger Z.D., Gopinathannair R. (2024). 2023 ACC/AHA/ACCP/HRS Guideline for the Diagnosis and Management of Atrial Fibrillation: A Report of the American College of Cardiology/American Heart Association Joint Committee on Clinical Practice Guidelines. Circulation.

[17] Osmancik P., Herman D., Neuzil P., Hala P., Taborsky M., Kala P., Poloczek M., Stasek J., Haman L., Branny M. (2020). Left Atrial Appendage Closure Versus Direct Oral Anticoagulants in High-Risk Patients With Atrial Fibrillation. J. Am. Coll. Cardiol..

[18] Boersma L.V., Ince H., Kische S., Pokushalov E., Schmitz T., Schmidt B., Gori T., Meincke F., Protopopov A.V., Betts T. (2019). Evaluating real-world clinical outcomes in atrial fibrillation patients receiving the WATCHMAN left atrial appendage closure technology. Circ. Arrhythm. Electrophysiol..

[19] Tzikas A., Shakir S., Gafoor S., Omran H., Berti S., Santoro G., Kefer J., Landmesser U., Nielsen-Kudsk J.E., Cruz-Gonzalez I. (2016). Left atrial appendage occlusion for stroke prevention in atrial fibrillation: Multicentre experience with the AMPLATZER cardiac plug. EuroIntervention.

[20] Korsholm K., Valentin J.B., Damgaard D., Diener H.C., Camm A.J., Landmesser U., Hildick-Smith D., Johnsen S.P., Nielsen-Kudsk J.E. (2022). Clinical outcomes of left atrial appendage occlusion versus direct oral anticoagulation in patients with atrial fibrillation and prior ischemic stroke: A propensity-score matched study. Int. J. Cardiol..

[21] Nielsen-Kudsk J.E., Korsholm K., Damgaard D., Valentin J.B., Diener H.C., Camm A.J., Johnsen S.P. (2021). Clinical Outcomes Associated With Left AtrialAppendageOcclusion Versus Direct OralAnticoagulation in AtrialFibrillation. JACC Cardiovasc. Interv..

[22] Price M.J., Slotwiner D., Du C., Freeman J.V., Turi Z., Rammohan C., Kusumoto F.M., Kavinsky C., Akar J., Varosy P.D. (2022). Clinical Outcomesat 1 Year Following Transcatheter Left AtrialAppendageOcclusion in the United States. JACC Cardiovasc. Interv..

[23] Friedman D.J., Du C., Wang Y., Agarwal V., Varosy P.D., Masoudi F.A., Holmes D.R., Reddy V.Y., Price M.J., Curtis J.P. (2022). Patient-Level Analysis of Watchman Left AtrialAppendageOcclusion in Practice Versus Clinical Trials. JACC Cardiovasc. Interv..

[24] Bonde A.N., Blanche P., Staerk L., Gerds T.A., Gundlund A., Gislason G., Torp-Pedersen C., Lip G.Y.H., Hlatky M.A., Olesen J.B. (2019). Oralanticoagulationamongatrialfibrillationpatients with anaemia: An observationalcohort study. Eur. Heart J..

[25] Larsen T.B., Skjøth F., Nielsen P.B., Kjældgaard J.N., Lip G.Y. (2016). Comparative effectiveness and safety of non-vitamin K antagonistoralanticoagulants and warfarin in patients with atrial fibrillation: Propensity weighted nationwide cohort study. BMJ.

[26] Van den Ham H.A., Souverein P.C., Klungel O.H., Platt R.W., Ernst P., Dell’Aniello S., Schmiedl S., Grave B., Rottenkolber M., Huerta C. (2021). Major bleeding in users of direct oral anticoagulants in atrial fibrillation: A pooled analysis of results from multiple population-based cohort studies. Pharmacoepidemiol. Drug Saf..

[27] Shatla I., El-Zein R.S., Kennedy K., Elkaryoni A., Ubaid A., Wimmer A.P. (2022). Comparison of the Safety of Left Atrial Appendage Occlusion in Patients Aged <75 Versus Those Aged ≥75 Years (from a Nationwide Cohort Sample). Am. J. Cardiol..

[28] Nielsen-Kudsk J.E., Johnsen S.P., Wester P., Damgaard D., Airaksinen J., Lund J., De Backer O., Pakarinen S., Odenstedt J., Vikman S. (2017). Left atrial appendage occlusion versus standard medical care in patients with atrial fibrillation and intracerebral haemorrhage: A propensity score-matched follow-up study. EuroIntervention.

[29] Schrag M., Mac Grory B., Nackenoff A., Eaton J., Mistry E., Kirshner H., Yaghi S., Ellis C.R. (2021). Left Atrial Appendage Closure for Patients with Cerebral Amyloid Angiopathy and Atrial Fibrillation: The LAA-CAA Cohort. Transl. Stroke Res..

[30] Revesz T., Holton J.L., Lashley T., Plant G., Rostagno A., Ghiso J., Frangione B. (2002). Sporadic and familial cerebral amyloid angiopathies. Brain Pathol..

[31] Triantafyllou K., Gkolfakis P., Gralnek I.M., Oakland K., Manes G., Radaelli F., Awadie H., Camus Duboc M., Christodoulou D., Fedorov E. (2021). Diagnosis and management of acute lowergastrointestinalbleeding: European Society of GastrointestinalEndoscopy (ESGE) Guideline. Endoscopy.

[32] Lempereur M., Aminian A., Freixa X., Gafoor S., Shakir S., Omran H., Berti S., Santoro G., Kefer J., Landmesser U. (2017). Left atrial appendage occlusion in patients with atrial fibrillation and previous major gastrointestinal gleeding (from the Amplatzer Cardiac Plug Multicenter Registry). Am. J. Cardiol..

[33] De Vriese A.S., Caluwé R., Pyfferoen L., De Bacquer D., De Boeck K., Delanote J., De Surgeloose D., Van Hoenacker P., Van Vlem B., Verbeke F. (2020). Multicenter Randomized Controlled Trial of Vitamin K Antagonist Replacement by Rivaroxaban with or without Vitamin K2 in Hemodialysis Patients with Atrial Fibrillation: The Valkyrie Study. J. Am. Soc. Nephrol..

[34] Pokorney S.D., Chertow G.M., Al-Khalidi H.R., Gallup D., Dignacco P., Mussina K., Bansal N., Gadegbeku C.A., Garcia D.A., Garonzik S. (2022). Apixaban for Patients With Atrial Fibrillation on Hemodialysis: A Multicenter Randomized Controlled Trial. Circulation.

[35] Reinecke H., Engelbertz C., Bauersachs R., Breithardt G., Echterhoff H.H., Gerß J., Haeusler K.G., Hewing B., Hoyer J., Juergensmeyer S. (2023). A Randomized Controlled Trial Comparing Apixaban With the Vitamin K Antagonist Phenprocoumon in Patients on Chronic Hemodialysis: The AXADIA-AFNET 8 Study. Circulation.

[36] Hindricks G., Potpara T., Dagres N., Arbelo E., Bax J.J., Blomström-Lundqvist C., Boriani G., Castella M., Dan G.A., Dilaveris P.E. (2021). 2020 ESC Guidelines for the diagnosis and management of atrial fibrillation developed in collaboration with the European Association for Cardio-Thoracic Surgery (EACTS): The Task Force for the diagnosis and management of atrial fibrillation of the European Society of Cardiology (ESC) Developed with the special contribution of the European Heart Rhythm Association (EHRA) of the ESC. Eur. Heart J..

[37] Luani B., Genz C., Herold J., Mitrasch A., Mitusch J., Wiemer M., Schmeißer A., Braun-Dullaeus R.C., Rauwolf T. (2019). Cerebrovascular events, bleeding complications and device related thrombi in atrial fibrillation patients with chronic kidney disease and left atrial appendage closure with the WATCHMAN™ device. BMC Cardiovasc. Disord..

[38] Kramer A.D., Korsholm K., Kristensen A., Poulsen L.H., Nielsen-Kudsk J.E. (2022). Left atrialappendageocclusion in haemophilia patients with atrial fibrillation. J. Interv. Card. Electrophysiol..

[39] Seiffge D.J., De Marchis G.M., Koga M., Paciaroni M., Wilson D., Cappellari M., Macha Md K., Tsivgoulis G., Ambler G., Arihiro S. (2020). RAF, RAF-DOAC, CROMIS-2, SAMURAI, NOACISP, Erlangen, and Verona registrycollaborators. Ischemic Stroke despiteOralAnticoagulant Therapy in Patients with AtrialFibrillation. Ann. Neurol..

[40] Freixa X., Cruz-González I., Regueiro A., Nombela-Franco L., Estévez-Loureiro R., Ruiz-Salmerón R., Bethencourt A., Gutiérrez-García H., Fernández-Díaz J.A., Moreno-Samos J.C. (2019). Left Atrial Appendage Occlusion as Adjunctive Therapy to Anticoagulation for Stroke Recurrence. J. Invasive Cardiol..

[41] Glikson M., Wolff R., Hindricks G., Mandrola J., Camm A.J., Lip G.Y.H., Fauchier L., Betts T.R., Lewalter T., Saw J. (2020). EHRA/EAPCI expert consensus statement on catheter-based left atrial appendage occlusion—An update. EuroIntervention.

[42] Marzec L.N., Wang J., Shah N.D., Chan P.S., Ting H.H., Gosch K.L., Hsu J.C., Maddox T.M. (2017). Influence of direct oral anticoagulants on rates of oral anticoagulation for atrial fibrillation. J. Am. Coll. Cardiol..

[43] Boersma L.V., Ince H., Kische S., Pokushalov E., Schmitz T., Schmidt B., Gori T., Meincke F., Protopopov A.V., Betts T. (2017). Efficacy and safety of left atrial appendage closure with WATCHMAN in patients with or without contraindication to oral anticoagulation: 1-Year follow-up outcome data of the EWOLUTION trial. Heart Rhythm.

[44] Reddy V.Y., Sievert H., Halperin J., Doshi S.K., Buchbinder M., Neuzil P., Huber K., Whisenant B., Kar S., Swarup V. (2014). Percutaneous left atrial appendage closure vs warfarin for atrial fibrillation: A randomized clinical trial. JAMA.

[45] Price M.J., Friedman D.J., Du C., Wang Y., Lin Z., Curtis J.P., Freeman J.V. (2022). Comparative Safety of Transcatheter LAAO With the First-Generation Watchman and Next-Generation Watchman FLX Devices. J. Am. Coll. Cardiovasc. Interv..

[46] Kar S., Doshi S.K., Sadhu A., Horton R., Osorio J., Ellis C., Stone J., Shah M., Dukkipati S.R., Adler S. (2021). PrimaryOutcome Evaluation of a Next-Generation Left AtrialAppendageClosure Device: Results From the PINNACLE FLX Trial. Circulation.

[47] Saliba W.I., Kawai K., Sato Y., Kopesky E., Cheng Q., Ghosh S.K.B., Herbst T.J., Kawakami R., Konishi T., Virmani R. (2023). Enhanced Thromboresistance and Endothelialization of a Novel Fluoropolymer-Coated Left Atrial Appendage Closure Device. JACC Clin. Electrophysiol..

[48] Freeman J.V., Varosy P., Price M.J., Slotwiner D., Kusumoto F.M., Rammohan C., Kavinsky C.J., Turi Z.G., Akar J., Koutras C. (2020). The NCDR Left AtrialAppendageOcclusionRegistry. J. Am. Coll. Cardiol..

[49] Asmarats L., Rodes-Cabau J. (2017). Percutaneous left atrial appendage closure: Current devices and clinical outcomes. Circ. Cardiovasc. Interv..

[50] Lakkireddy D., Thaler D., Ellis C.R., Swarup V., Sondergaard L., Carroll J., Gold M.R., Hermiller J., Diener H.C., Schmidt B. (2021). Amplatzer amulet left atrial appendage occluder versus Watchman device for stroke prophylaxis (Amulet IDE): A randomized, controlled trial. Circulation.

[51] Galea R., De Marco F., Meneveau N., Aminian A., Anselme F., Gräni C., Huber A.T., Teiger E., Iriart X., Babongo Bosombo F. (2022). Amulet or Watchman device for percutaneous left atrial appendage closure: Primary results of the SWISS-APERO randomized clinical trial. Circulation.

[52] Pivato C.A., Liccardo G., Sanz-Sanchez J., Pelloni E., Pujdak K., Xuareb R.G., Cruz-Gonzalez I., Pisano F., Scotti A., Tarantini G. (2022). Left atrial appendage closure with the II generation Ultraseal device: An international registry. The LIGATE study. Catheter. Cardiovasc. Interv..

[53] Wilkins B., Srimahachota S., De Backer O., Boonyartavej S., Lertsuwunseri V., Tumkosit M., Søndergaard L. (2021). First-in-human results of the Omega leftatrialappendage occluder for patients with non-valvularatrialfibrillation. EuroIntervention.

[54] Chow D.H.F., Wong Y.H., Park J.W., Lam Y.Y., De Potter T., Rodés-Cabau J., Asmarats L., Sandri M., Sideris E., McCaw T. (2019). An overview of current and emerging devices for percutaneous left atrial appendage closure. Trends Cardiovasc. Med..

[55] De Backer O., Hafiz H., Fabre A., Lertsapcharoen P., Srimahachota S., Foley D., Sondergaard L. (2021). State-of-the-art preclinical testing of the OMEGA^TM^ leftatrialappendage occluder. Catheter. Cardiovasc. Interv..

[56] Huang H., Liu Y., Xu Y., Wang Z., Li Y., Cao K., Zhang S., Yang Y., Yang X., Huang D. (2017). Percutaneous left atrial appendage closure with the LAmbre device for stroke prevention in atrial fibrillation: A prospective, multicenter clinical study. JACC Cardiovasc. Interv..

[57] Sommer R.J., Lamport R., Melanson D., Devellian C., Levine A., Cain C.M., Kaplan A.V., Gray W.A. (2021). Preclinical assessment of a novel conformable foam-based left atrial appendage closure device. Biomed. Res. Int..

[58] Sommer R.J., Kim J.H., Szerlip M., Chandhok S., Sugeng L., Cain C., Kaplan A.V., Gray W.A. (2021). Conformal Left Atrial Appendage Seal Device for Left Atrial Appendage Closure. J. Am. Coll. CardiolIntv..

[59] Wong G.X., Kar S., Smith T.W., Spangler T., Bolling S.F., Rogers J.H. (2023). Transcatheter Left Atrial Appendage Exclusion: Preclinical and Early Clinical Results With the Laminar Device. J. Am. Coll. Cardiol. Interv..

[60] Bavishi C. (2023). Transcatheter Left Atrial Appendage Closure: Devices Available, Pitfalls, Advantages, and Future Directions. US Cardiol. Rev..

[61] Fukutomi M., Fuchs A., Bieliauskas G., Wong I., Kofoed K.F., Søndergaard L., De Backer O. (2022). Computed tomography-based selection of transseptal puncture site for percutaneous left atrial appendage closure. EuroIntervention.

[62] Wang D.D., Eng M., Kupsky D., Myers E., Forbes M., Rahman M., Zaidan M., Parikh S., Wyman J., Pantelic M. (2016). Application of 3-Dimensional Computed Tomographic Image Guidance to WATCHMAN Implantation and Impact on Early Operator Learning Curve: Single-Center Experience. JACC Cardiovasc. Interv..

[63] Galea R., Räber L., Fuerholz M., Häner J.D., Siontis G.C.M., Brugger N., Moschovitis A., Heg D., Fischer U., Meier B. (2021). Impact of Echocardiographic Guidance on Safety and Efficacy of Left Atrial Appendage Closure: An Observational Study. JACC Cardiovasc. Interv..

[64] Lennon M.J., Gibbs N.M., Weightman W.M., Leber J., Ee H.C., Yusoff I.F. (2005). Transesophageal echocardiographyrelated gastrointestinal complications in cardiac surgical patients. J. Cardiothorac. Vasc. Anesth..

[65] Berti S., Pastormerlo L.E., Santoro G., Brscic E., Montorfano M., Vignali L., Danna P., Tondo C., Rezzaghi M., D’Amico G. (2018). Intracardiac Versus Transesophageal Echocardiographic Guidance for Left Atrial Appendage Occlusion: The LAAO Italian Multicenter Registry. JACC Cardiovasc. Interv..

[66] Nielsen-Kudsk J.E., Berti S., De Backer O., Aguirre D., Fassini G., Cruz-Gonzalez I., Grassi G., Tondo C. (2019). Use of Intracardiac Compared With Transesophageal Echocardiography for Left Atrial Appendage Occlusion in the Amulet Observational Study. JACC Cardiovasc. Interv..

[67] Alkhouli M., Chaker Z., Alqahtani F., Raslan S., Raybuck B. (2020). Outcomes of Routine Intracardiac Echocardiography to Guide Left Atrial Appendage Occlusion. JACC Clin. Electrophysiol..

[68] Ribeiro J.M., Teixeira R., Puga L., Costa M., Gonçalves L. (2019). Comparison of intracardiac and transoesophageal echocardiography for guidance of percutaneous left atrial appendage occlusion: A meta-analysis. Echocardiography.

[69] Nielsen-Kudsk J.E., Berti S., Caprioglio F., Ronco F., Arzamendi D., Betts T., Tondo C., Christen T., Allocco D.J. (2023). Intracardiac Echocardiography to Guide Watchman FLX Implantation: The ICE LAA Study. JACC Cardiovasc. Interv..

[70] Alkhouli M., Simard T., El Shaer A., Bird J., Nkomo V.T., Freidman P.A., Thaden J., Padang R. (2022). First Experience With a Novel Live 3D ICE Catheter to Guide Transcatheter Structural Heart Interventions. JACC Cardiovasc. Imaging..

[71] Dukkipati S.R., Kar S., Holmes D.R., Doshi S.K., Swarup V., Gibson D.N., Maini B., Gordon N.T., Main M.L., Reddy V.K. (2018). Device-related thrombus after left atrial appendage closure. Circulation.

[72] Alkhouli M., Busu T., Shah K., Osman M., Alqahtani F., Raybuck B. (2018). Incidence and clinical impact of device-related thrombus following percutaneous left atrial appendage occlusion: A meta-analysis. J. Am. Coll. Cardiol. EP.

[73] Korsholm K., Jensen J.M., Nørgaard B.L., Nielsen-Kudsk J.E. (2019). Detection of Device-Related Thrombosis Following Left Atrial Appendage Occlusion: A Comparison Between Cardiac Computed Tomography and Transesophageal Echocardiography. Circ. Cardiovasc. Interv..

[74] Simard T., Jung R.G., Lehenbauer K., Piayda K., Pracoń R., Jackson G.G., Flores-Umanzor E., Faroux L., Korsholm K., Chun J.K.R. (2021). Predictors of Device-Related Thrombus Following Percutaneous Left Atrial Appendage Occlusion. J. Am. Coll. Cardiol..

[75] Samaras A., Papazoglou A.S., Balomenakis C., Bekiaridou A., Moysidis D.V., Patsiou V., Orfanidis A., Giannakoulas G., Kassimis G., Fragakis N. (2024). Residual leaks following percutaneous left atrial appendage occlusion and outcomes: A meta-analysis. Eur. Heart J..

[76] Clemente A., Avogliero F., Berti S., Paradossi U., Jamagidze G., Rezzaghi M., Della Latta D., Chiappino D. (2015). Multimodality imaging in preoperative assessment of left atrial appendage transcatheter occlusion with the Amplatzer Cardiac Plug. Eur. Heart J. Cardiovasc. Imaging.

[77] Korsholm K., Jensen J.M., Nørgaard B.L., Samaras A., Saw J., Berti S., Tzikas A., Nielsen-Kudsk J.E. (2021). Peridevice Leak Following Amplatzer Left Atrial Appendage Occlusion: Cardiac Computed Tomography Classification and Clinical Outcomes. JACC Cardiovasc. Interv..

[78] Alkhouli M., Du C., Killu A., Simard T., Noseworthy P.A., Friedman P.A., Curtis J.P., Freeman J.V., Holmes D.R. (2022). Clinical Impact of Residual Leaks Following Left Atrial Appendage Occlusion: Insights From the NCDR-LAAO Registry. JACC Clin. Electrophysiol..

